# Five advanced chatbots solving European Diploma in Radiology (EDiR) text-based questions: differences in performance and consistency

**DOI:** 10.1186/s41747-025-00591-0

**Published:** 2025-08-19

**Authors:** Jakub Pristoupil, Laura Oleaga, Vanesa Junquero, Cristina Merino, Suha Sureyya Ozbek, Lukas Lambert, European Society of Radiology (ESR)

**Affiliations:** 1 Department of Imaging Methods, Motol University Hospital and Second Faculty of Medicine, Charles University, Prague, Czech Republic; 2 Department of Radiology, Clinical Diagnostic Imaging Centre, Hospital Clínic de Barcelona, Barcelona, Spain; 3 European Board of Radiology, Av. Diagonal, 383, L’Eixample, 08008, Barcelona, Spain; 4 Era Radiology Center, Izmir, Turkey; 5 Am Gestade 1, Vienna, Austria

**Keywords:** Artificial intelligence, Education (medical), Educational measurement, European Diploma in Radiology, Radiology

## Abstract

**Background:**

We compared the performance, confidence, and response consistency of five chatbots powered by large language models in solving European Diploma in Radiology (EDiR) text-based multiple-response questions.

**Methods:**

ChatGPT-4o, ChatGPT-4o-mini, Copilot, Gemini, and Claude 3.5 Sonnet were tested using 52 text-based multiple-response questions from two previous EDiR sessions in two iterations. Chatbots were prompted to evaluate each answer as correct or incorrect and grade its confidence level on a scale of 0 (not confident at all) to 10 (most confident). Scores per question were calculated using a weighted formula that accounted for correct and incorrect answers (range 0.0–1.0).

**Results:**

Claude 3.5 Sonnet achieved the highest score per question (0.84 ± 0.26, mean ± standard deviation) compared to ChatGPT-4o (0.76 ± 0.31), ChatGPT-4o-mini (0.64 ± 0.35), Copilot (0.62 ± 0.37), and Gemini (0.54 ± 0.39) (*p* < 0.001). A self-reported confidence in answering the questions was 9.0 ± 0.9 for Claude 3.5 Sonnet followed by ChatGPT-4o (8.7 ± 1.1), compared to ChatGPT-4o-mini (8.2 ± 1.3), Copilot (8.2 ± 2.2), and Gemini (8.2 ± 1.6, *p* < 0.001). Claude 3.5 Sonnet demonstrated superior consistency, changing responses in 5.4% of cases between the two iterations, compared to ChatGPT-4o (6.5%), ChatGPT-4o-mini (8.8%), Copilot (13.8%), and Gemini (18.5%). All chatbots outperformed human candidates from previous EDiR sessions, achieving a passing grade from this part of the examination.

**Conclusion:**

Claude 3.5 Sonnet exhibited superior accuracy, confidence, and consistency, with ChatGPT-4o performing nearly as well. The variation in performance among the evaluated models was substantial.

**Relevance statement:**

Variation in performance, consistency, and confidence among chatbots in solving EDiR test-based questions highlights the need for cautious deployment, particularly in high-stakes clinical and educational settings.

**Key Points:**

- Claude 3.5 Sonnet outperformed other chatbots in accuracy and response consistency.
- ChatGPT-4o ranked second, showing strong but slightly less reliable performance.
- All chatbots surpassed EDiR candidates in text-based EDiR questions.

**Graphical Abstract:**

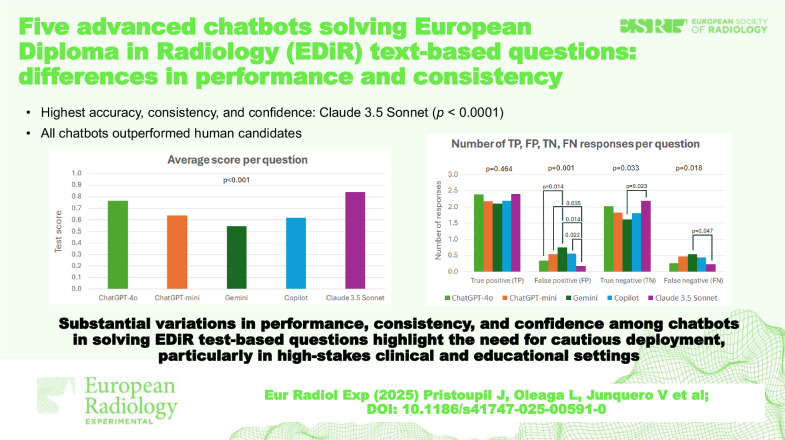

## Background

Chatbots are advanced conversational agents powered by large language models (LLMs) that have gained increasing attention in the field of medical education, data interpretation, analysis and translation of medical reports, structured reporting, and decision-making [1–5]. While chatbots have shown promise in various medical applications, their ability to accurately handle medical examination questions, particularly in specialized fields like radiology, remains underexplored in models other than ChatGPT [6–8].

The European Diploma in Radiology (EDiR) represents a standardized assessment of radiological knowledge and competence [9, 10]. With growing interest in AI-powered tools, their utility is being explored even in medical education. Understanding how different chatbot systems perform in handling specialized examination questions becomes insightful. While these AI systems have shown capability in processing text-based queries, they currently have limited ability to analyze medical imaging data, making text-based questions an ideal starting point for evaluating their performance in medical assessment scenarios [11, 12].

This study aims to compare the performance and consistency of leading-edge LLMs in solving text-based multiple-response questions from the EDiR. By analyzing their responses, this research seeks to identify the most reliable and effective chatbot for reinforcing radiologists’ professional knowledge.

## Methods

Ethical approval for this study was obtained from the Ethics Committee of the General University Hospital in Prague (79/24 S-IV Grant). Informed consent was not required.

### Test questions

The EDiR is a standardized international benchmark for general radiology that tests knowledge, skills, and competence in three components. Multiple-response questions section tests predominantly theoretical knowledge. Short cases section evaluates candidates’ ability to analyze clinical scenarios accompanied by imaging studies under time constraints. Clinically oriented reasoning evaluation comprises comprehensive clinical cases with detailed imaging data that test higher-order thinking and decision-making. Scoring of the exam and the required pass marks are based on specific rules and calculations that are beyond the scope of this article [9, 10, 13].

Test questions in this study were selected from two official EDiR sessions across Europe that took place in 2023. Only text-based multiple-response questions without any visual prompts were included. The number of questions was 52. Each question featured 5 responses from which one to four were correct. The score awarded per question was calculated as (“correct responses” / “all correct responses”) - (“incorrect responses” / “all incorrect responses”). The maximum score for a question was 1.0, and the minimum was 0.0. For example, if 1 of 2 correct options and 1 of 3 incorrect options were selected, the score was 0.17 (1/2–1/3). If 2 of 4 correct options and 1 of 1 incorrect options were selected, the score was 0.0 (2/4-1/1, minimum score 0.0). Ground truth and scores from EDiR candidates were provided by the administrator.

### Chatbots and models

Internet search and preliminary testing revealed five chatbots that were suitable for solving EDiR test questions:

- *ChatGPT-4o*, a generative AI model based on GPT-4 LLM developed by OpenAI (San Francisco, CA, USA) and released in May 2024, designed for advanced natural language processing and conversational tasks;
- *ChatGPT-4o-mini*, a streamlined, less complex version of ChatGPT-4o, which is available at no cost;
- *Gemini*, a generative chatbot based on LLM of the same name, which was developed by Google (Mountain View, CA, USA) and released in March 2023 since then undergoing development (the model used in the study was 1.5 released in May 2024); in this study, we used the 1.5 Flash model, which integrates Internet search;
- *Copilot*, a generative chatbot developed by Microsoft (Redmond, WA, USA) and introduced in February 2023, is based on GPT-4 LLM and utilizes the Microsoft Prometheus model that iteratively generates search queries using Bing Search (Microsoft) Internet search and GPT-4;
- *Claude 3.5 Sonnet*, the last tested generative chatbot, developed by Antrophic (San Francisco, CA, USA) and released in June 2024; it uses an LLM of the same name (upgrading to the paid version that we used substantially increases the number of prompts that can be processed).

### Generating prompts

For each question, a new prompt was used to reset the context. The prompt was composed of the following quotations:

“*This is a test question from a test for radiologists*

*In each option, please state if the answer is correct or incorrect, how confident the answer is (on a scale 0 to 10, with 10 meaning most confident and 0 not confident at all)*.

*Conclude with a summary of evaluations per item (correct/incorrect - confidence).*”

Followed by the question as shown in the test interface: Example (mock question due to confidentiality agreement):

“*Regarding staging of rectal cancer: Which of the following statements are correct?*

*(Multiple answers might be correct)*

1. *Computed tomography should be used for local staging*.
2. *Perirectal lymph nodes above 10* *mm in short axis should be reported as suspicious*.
3. *TNM T3 tumors extend into the mesorectal fat*.
4. *Muscularis propria has low signal intensity on T2-weighted images*.
5. *Local staging requires intravenous contrast material.”*

The confidence was self-reported by each of the chatbots on a scale from 0 (not confident at all) to 10 (most confident) for every answer to each question. Each question was presented to each model during two iterations between October 20 and 24, 2024 (first iteration) and between November 1 and 6, 2024 (second iteration) by one of the authors (J.P.). The chatbots did not receive any feedback whether their responses were correct or not.

### Statistical analysis

Statistical analysis was performed in R (R Foundation, Vienna, Austria) and Prism (GraphPad, La Jolla, CA, USA). The distribution of the data was assessed using the D’Agostino-Person omnibus test and visual inspection of the data histograms. The variables were expressed as mean ± standard deviation (SD). Comparison among the chatbots was performed using analysis of variance (ANOVA) with Tukey’s *post hoc* tests. The significance of changes in responses between the first and second iterations (model consistency) was assessed using Fisher’s exact test. Interobserver agreement was expressed as Fleiss κ (“Library irr” R package), and 95% confidence intervals were constructed by bootstrapping (“Library boot” R package). A *p*-value below 0.050 was considered statistically significant.

## Results

**Fig. 1:**
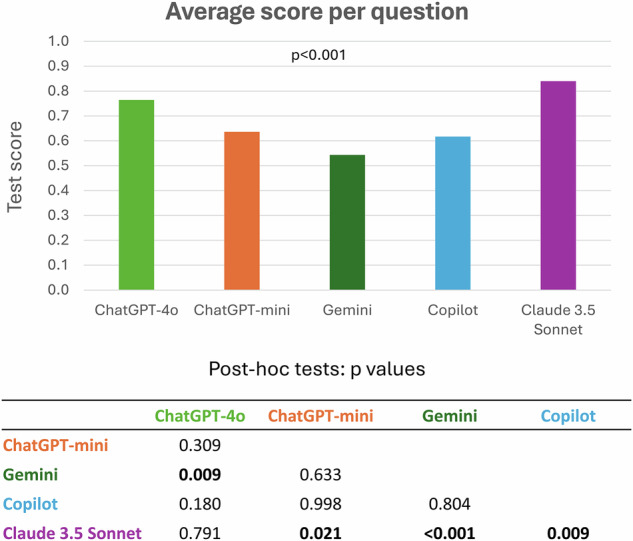
Comparison of average score per question among five large language models

**Fig. 2:**
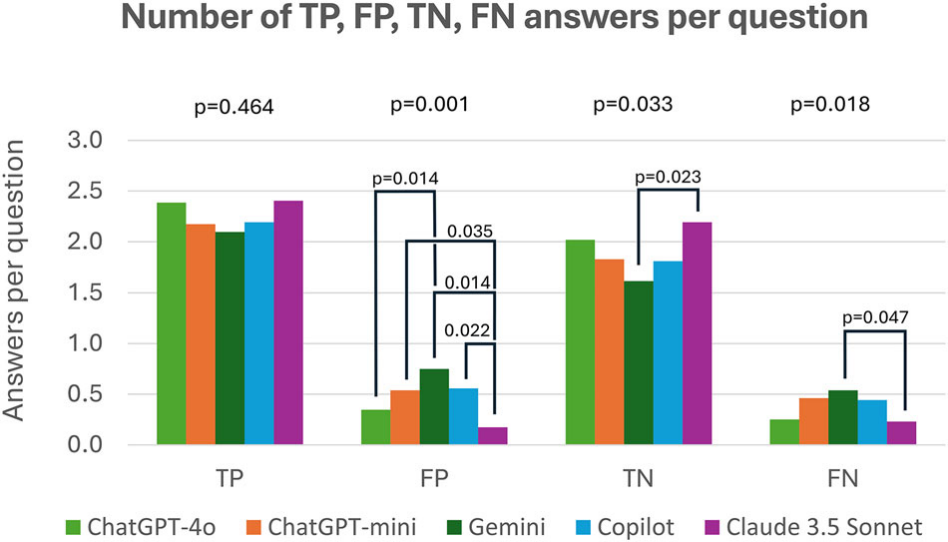
Comparison of the average number of true positives (TP), false positives (FP), true negatives (TN), and false negatives (FN) answers per question among five large language models (ChatGPT-4o, ChatGPT-mini, Gemini, Copilot, Claude 3.5 Sonnet)

**Fig. 3:**
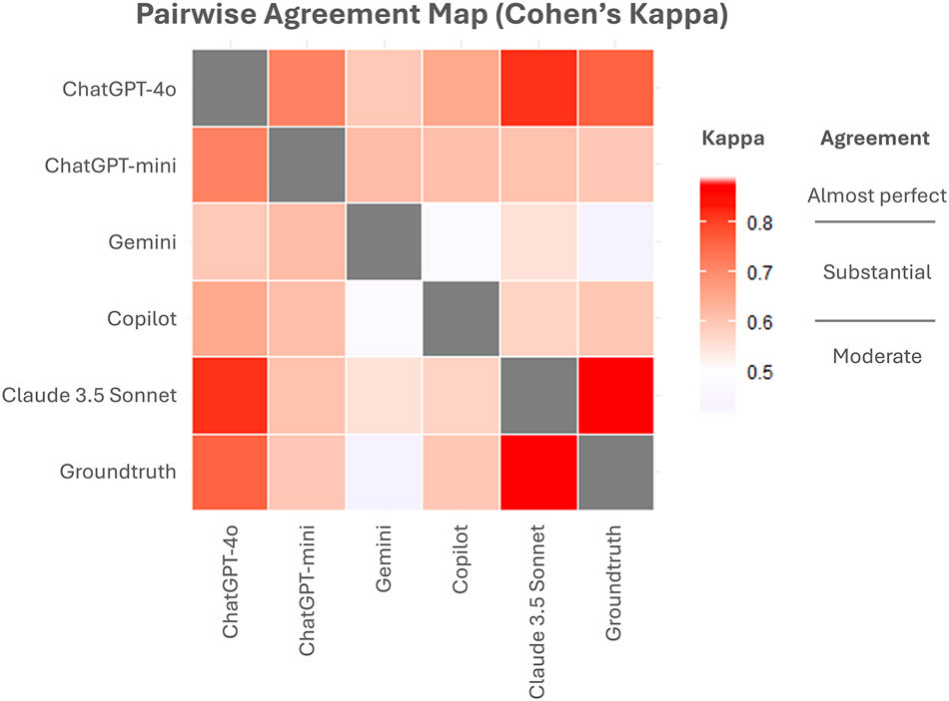
Agreement among large language models and ground truth

**Fig. 4:**
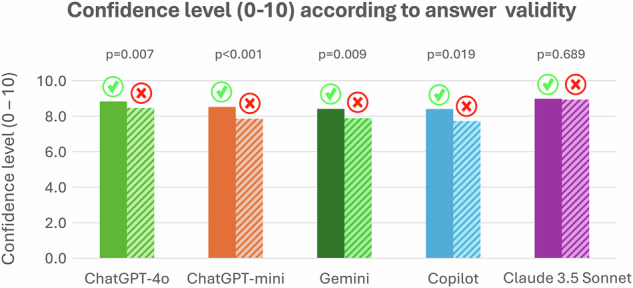
Self-reported confidence (from 0 = not confident at all to 10 = most confident) of large language models according to validity (tick, correct answer; cross, incorrect answer)

All models were able to answer all questions and give confidence ratings. The best performance was achieved by Claude 3.5 Sonnet (score per question, 0.84 ± 0.26, mean ± standard deviation) followed by ChatGPT-4o (0.76 ± 0.31), ChatGPT-4o-mini (0.64 ± 0.35), Copilot (0.62 ± 0.37), and Gemini (0.54 ± 0.39, overall *p* < 0.0001, Figs. 1 and 2, Table 1). EDiR candidates (*n* = 42) scored on average 0.49 ± 0.10 per question. The agreement among the LLMs ranged from 0.49 (Gemini and Copilot) to 0.81 (ChatGPT-4o and Claude 3.5 Sonnet, Fig. 3). A self-reported confidence in answering the questions was 9.0 ± 0.9 for Claude 3.5 Sonnet and 8.7 ± 1.1 for ChatGPT-4o, followed by ChatGPT-4o-mini (8.2 ± 1.3), Copilot (8.2 ± 2.2), and Gemini (8.2 ± 1.6, overall *p* < 0.0001, *post hoc p*-values in Supplementary Table S1). Apart from Claude 3.5 Sonnet, all other LLMs reported significantly greater confidence in responses that they answered correctly (Fig. 4). The average confidence level in correct responses across all models was 8.6 ± 1.3 compared to 8.2 ± 1.8 in incorrect responses (*p* < 0.001). When comparing responses between the first and the second iterations, Claude 3.5 Sonnet showed the best consistency, changing responses in 14 of 260 answers (5.4%), compared to Gemini, with the highest number of changes in 48 of 260 answers (18.5%) (Table 2, Supplemental Fig. S1). The confidence in answering questions dramatically increased in the second iteration in Gemini (*p* < 0.0001), slightly increased in Claude 3.5 Sonnet (*p* = 0.001), and decreased in ChatGPT-4o (*p* = 0.002) and ChatGPT-4o-mini (*p* < 0.001, Table 3).

**Table 1:** Average score per question, number of correct responses (260 responses), and questions with all correct responses (52 questions)

Model	Score per question Mean ± standard deviation	Correct responses Number (%)	Questions with all correct responses Number (%)
ChatGPT-4o	0.76 ± 0.31	229 (88%)	28 (54%)
ChatGPT-mini	0.64 ± 0.35	208 (80%)	18 (35%)
Gemini	0.54 ± 0.39	193 (74%)	16 (31%)
Copilot	0.62 ± 0.37	208 (80%)	19 (37%)
Claude 3.5 Sonnet	0.84 ± 0.26	239 (92%)	35 (67%)

**Table 2:** Comparison of responses in the first and second run in terms of agreement, number of changed responses (5 answers in 52 questions = 260 responses), and scores per question

Model	Agreement between 1st and 2nd run κ (95% CI)	Changed answers Number (%)	*p*-value	Score per question (1st run) Mean ± SD	Score per question (2nd run) Mean ± SD	*p*-value
ChatGPT-4o	0.88 (0.81–0.93)	17 (6.5%)	0.294	0.76 ± 0.31	0.74 ± 0.31	0.559
ChatGPT-mini	0.82 (0.75–0.88)	23 (8.8%)	0.155	0.64 ± 0.35	0.61 ± 0.36	0.522
Gemini	0.63 (0.53–0.72)	48 (18.5%)	< 0.001	0.54 ± 0.39	0.53 ± 0.37	0.723
Copilot	0.72 (0.63–0.81)	36 (13.8%)	0.027	0.62 ± 0.37	0.62 ± 0.38	0.925
Claude 3.5 Sonnet	0.89 (0.83–0.95)	14 (5.4%)	0.391	0.84 ± 0.26	0.84 ± 0.28	1.000

*CI* Confidence interval, *SD* Standard deviation

**Table 3:** Comparison of confidence levels (0 = not confident; 10 = most confident) per response

Model	Confidence level (1st run) Mean ± SD	Confidence level (2nd run) Mean ± SD	*p*-value
ChatGPT-4o	8.7 ± 1.1	8.5 ± 1.0	0.002
ChatGPT-mini	8.2 ± 1.3	8.0 ± 1.5	< 0.001
Gemini	8.2 ± 1.6	9.1 ± 1.4	< 0.001
Copilot	8.2 ± 2.2	8.0 ± 2.9	0.350
Claude 3.5 Sonnet	9.0 ± 0.9	9.1 ± 0.83	0.001

*SD* Standard deviation

## Discussion

In this study, we showed that Claude 3.5 Sonnet provided the best scores with the highest confidence, with ChatGPT-4o as a runner-up. Claude excelled especially providing less false positive answers. All models overall expressed a high level of confidence, which, except for Claude 3.5 Sonnet, was lower in incorrectly answered questions.

The differences among examined chatbots in solving EDiR questions demonstrate different capabilities of LLMs in distinguishing true a false statements about highly specific medical knowledge. The superior performance of Claude 3.5 Sonnet and ChatGPT-4o aligns with findings by several research groups. Savage et al [14] and Keshavarz et al [15] demonstrated that newer generations of LLMs show marked improvements in medical knowledge assessment compared to their predecessors. The almost perfect agreement between Claude 3.5 Sonnet and ChatGPT-4o demonstrates that leading LLMs are developing consistent knowledge representations of radiological concepts, though the significantly lower performance of other models indicates that this capability is not universal across all AI systems. Previous studies have consistently demonstrated ChatGPT’s superior performance across various medical specialties, including radiology [6, 8], while reports on Claude 3.5 Sonnet, which has now exhibited overall superior performance, have been limited.

It should be noted that much of the existing research has not explored open-ended questions, where chatbots may exhibit a tendency to provide suggestive yet incorrect answers. This can potentially lead to confusion or misunderstanding, especially for individuals lacking sufficient expertise in the relevant medical specialty.

Compared to results from live EDiR sessions with EDiR candidates, all systems were superior to the performance of all participants, meaning that they would reach a passing grade if image-based questions were not considered. This is an advancement compared to the pioneering work by Bhayana et al [7], where ChatGPT 3.5 answered 69% of single-best answer questions correctly. Compared to single-best-answer questions, multiple-response questions are regarded as more demanding and are associated with lower scores [16].

In past LLM research of chatbots solving medical licensing test questions, ChatGPT has been exhibiting the best overall accuracy compared to other LLMs [17, 18]. Although ChatGPT may show greater accuracy in its responses to frequently asked questions in breast radiology, Gemini and Copilot score higher on readability scales [19]. Recently, ChatGPT, Copilot, Gemini, and Claude 3.5 Sonnet have been compared in solving neuroscience United States Medical Licensing Examination (USMLE) style test questions, with Claude 3.5 Sonnet showing the best results and ChatGPT as a runner-up by a small difference [20]. While Claude 3.5 Sonnet demonstrates superior performance in radiological knowledge, other systems with lower scores may be better suited for different applications. Although Copilot and both versions of ChatGPT are based on the same GPT-4 LLM technology, their outputs differ in various aspects. This suggests that optimizing chatbots for specific purposes impacts their performance in particular tasks and underscores the need for testing before selecting the most suitable one.

A particularly noteworthy finding is the relationship between confidence levels and accuracy among different models. While most chatbots showed lower confidence in incorrect answers, the best-performing Claude 3.5 Sonnet maintained consistent high confidence regardless of answer accuracy. This pattern contradicts observations by Omar et al [21] in their analysis of LLM performance in medical examinations, where they found that worse-performing models tend to exhibit “overconfidence” in medical knowledge. This raises important considerations about the reliability of self-reported confidence metrics in AI systems and their potential impact on medical education applications. In our study, LLMs demonstrated a high level of self-confidence, even when providing incorrect responses. LLMs are known to lack critical judgment regarding their outputs, and this phenomenon is similar to what has been referred to as “hallucinations” or “dogmatism.” Therefore, qualified oversight of their outputs remains essential [22].

The consistency of responses between iterations varied significantly among the tested models, with Claude 3.5 Sonnet showing the highest consistency (5.4% changed answers) compared to Gemini’s more variable performance (18.5% changed answers). Variations in response consistency have been reported by Funk et al [23] in their comparison of ChatGPT 3.5 and ChatGPT 4 for solving medical questions, suggesting that this remains a crucial area for improvement in weaker AI systems. The observed increase in confidence levels across iterations for some models, particularly Gemini, raises questions about the underlying mechanisms of confidence calibration in these systems. These effects can likely be attributed to several factors, including differences in the size and scope of the training datasets, model design, including the integration of online search capabilities, and the tradeoff between the model’s versatility and its complexity. Additionally, it is assumed that the models did not derive any inferences from their previous responses, as the context window was consistently reset after each interaction. If the context is reset, the influence of previous interaction is completely erased.

The high performance of leading chatbots in handling text-based radiological questions suggests potential applications in medical education, particularly for self-assessment and exam preparation. However, as noted by several researchers [11, 12], the limited performance of current LLMs to process medical imaging data remains a significant constraint. The high performance achieved by Claude 3.5 Sonnet and ChatGPT-4o in this study, while promising, should be considered within the context of these limitations and the specific nature of text-based multiple-response questions, which represent only one aspect of radiological knowledge assessment. Additionally, it should be noted that LLMs have also been employed in the preparation of exam tasks and the automated evaluation of free-text response questions [3]. Despite the widespread use of artificial intelligence in radiology, radiologists are expected to bear the responsibility for the accuracy and implications of AI-generated outputs [24].

The exceptional performance of LLMs in lower-order thinking questions challenges the traditional recall-based assessment format, underscoring the need to prioritize higher-order problem-solving skills that integrate both knowledge and experience. While a strong knowledge base remains fundamental in radiology—since one cannot identify what one does not recognize—greater emphasis should be placed on clinical reasoning, the practical application of acquired knowledge, and the synthesis of clinical and imaging findings and inputs generated by AI tools within specific diagnostic contexts. The Clinically Oriented Reasoning Evaluation and Short Cases modules of the EDiR exemplify this progressive approach, fostering a more comprehensive and clinically relevant assessment of radiological competence.

Several limitations of this study should be acknowledged. First, this study evaluated the performance of chatbots using text-based exam questions that assess lower-order cognitive skills. While multimodal chatbots are capable of processing general visual prompts, their ability to interpret medical images still remains limited [12]. For such tasks, specialized AI tools have been developed to analyze specific radiology examinations (chest x-ray, mammography, chest CT) for a narrow set of common diagnoses. Second, we did not evaluate the objective reasoning process underlying chatbots’ responses, which is an important aspect of their decision-making pathway. Third, the study did not investigate whether shuffling the answer choices in questions might influence the performance of the chatbots. Fourth, although a new prompt was generated for each question and no feedback was provided to the models, the possibility of cross-learning cannot be entirely ruled out. Fifth, the test questions were provided under a nondisclosure agreement, and they are not publicly available. However, we cannot fully account for sporadic leaks of test question fragments from past participants, which could provide an advantage to chatbots. Sixth, although the LLMs’ core designs were unchanged, Copilot and Gemini leveraged the dynamic and ever-evolving nature of the Internet. Finally, chatbots are continuously evolving, and this study represents an assessment of their current capabilities, which are subject to change as the technology advances.

In conclusion, this study highlights significant differences in the performance of LLMs when addressing text-based radiology examination questions within the EDiR framework. Among the evaluated models, Claude 3.5 Sonnet demonstrated the highest proficiency, exhibiting superior accuracy, confidence, and consistency, with ChatGPT-4o performing nearly as well. The observed variation in model performance underscores the importance of cautious deployment of chatbots, particularly in high-stakes clinical and educational settings.

## Supplementary information

**Additional file 1: Table S1.** *Post hoc* comparison of chatbot confidence levels in answering test questions (*p*-values). **Fig. S1.** Sankey plot showing changes in reponses (260 responses) between the 1st and 2nd run.

## Data Availability

The test questions utilized in this study are subject to a nondisclosure agreement. However, summary data can be obtained from the authors upon reasonable request and with approval from the European Board of Radiology.

## Abbreviations

AI: Artificial intelligence
EDiR: European Diploma in Radiology
GPT: Generative pretrained transformer
LLMs: Large language models
SD: Standard deviation
