# Supplementary material for: Five advanced chatbots solving European Diploma in Radiology (EDiR) text-based questions: differences in performance and consistency
Source: Eur Radiol Exp. 2025 Aug 19;9:79. doi: 10.1186/s41747-025-00591-0 (PMC12364795; doi:10.1186/s41747-025-00591-0)

Five advanced chatbots solving European Diploma in Radiology (EDiR) text-based questions: differences in performance and consistency

ELECTRONIC SUPPLEMENTARY MATERIAL

Table S1. *Post hoc* comparison of chatbot confidence levels in answering test questions (*p*-values).

	ChatGPT-mini *p*-value	Gemini *p*-value	Copilot *p*-value	Claude 3.5 Sonnet *p*-value
ChatGPT-4o	0.005	0.001	<0.001	0.204
ChatGPT-mini	x	0.985	0.936	<0.001
Gemini		x	0.999	<0.001
Copilot			x	<0.001

Fig. S1. Sankey plot showing changes in reponses (260 responses) between the 1st and 2nd run.

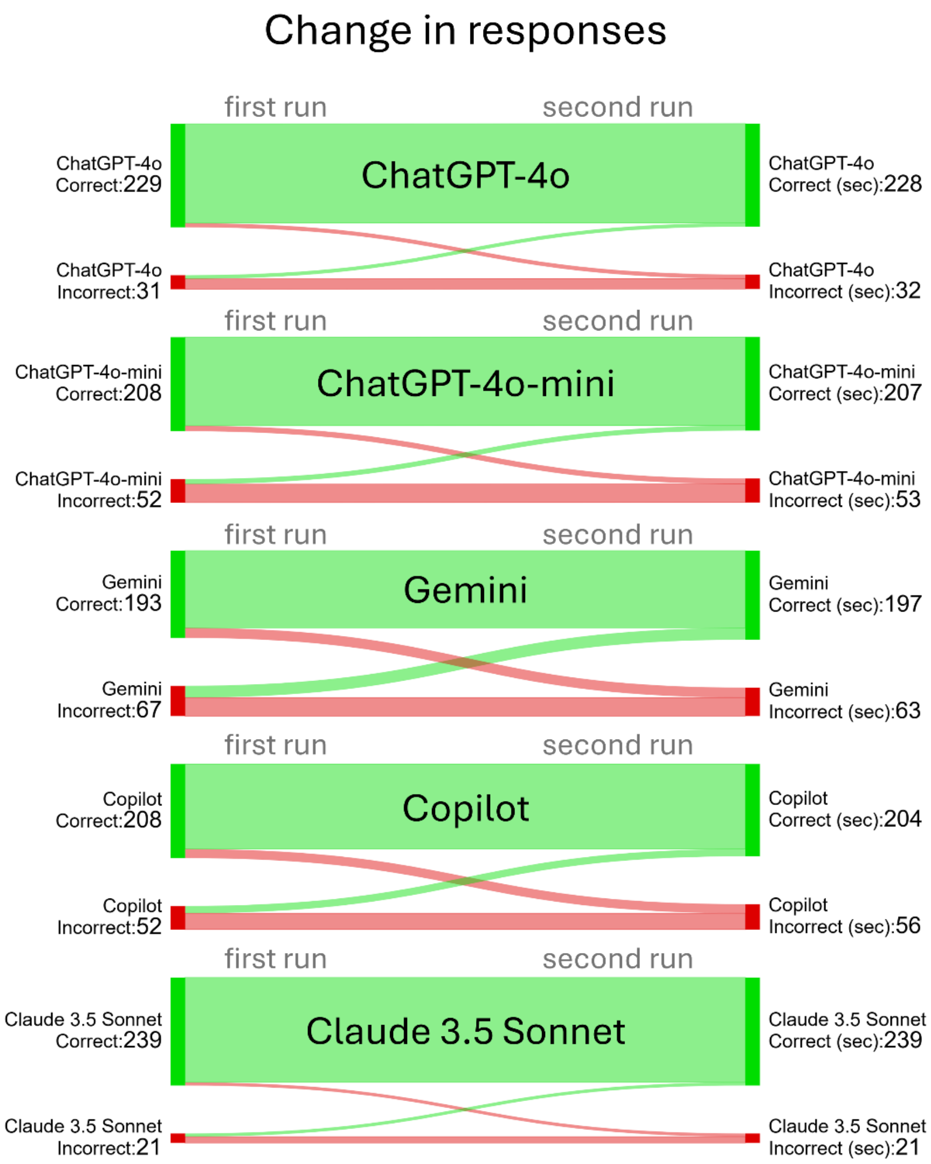

Supplement: Supplementary file 1 — Additional file 1: Table S1. Post hoc comparison of chatbot confidence levels in answering test questions (p-values). Fig. S1. Sankey plot showing changes in reponses (260 responses) between the 1st and 2nd run. [file 41747_2025_591_MOESM1_ESM.pdf]
